# Effects of probiotics combined with statins on blood lipids, TMAO, and inflammatory and oxidative stress markers in patients with atherosclerosis: a systematic review and meta-analysis

**DOI:** 10.3389/fcvm.2026.1897708

**Published:** 2026-08-13

**Authors:** Jie Dong, Lei Chen, Nan Zheng, Ming Yang

**Affiliations:** Department of Cardiovascular Surgery Institute of Cardiac Surgery, The Sixth Medical Center of PLA General Hospital, Beijing, China

**Keywords:** atherosclerosis, plaque stability, probiotics, statins, trimethylamine N-oxide

## Abstract

**Background:**

This systematic review and meta-analysis evaluated the effects of combining probiotics with statins on blood lipids, TMAO, and plaque stability in atherosclerotic patients.

**Methods:**

Literature was systematically searched in PubMed, Cochrane Library, Embase, and Web of Science through April 2026. Eligible studies were assessed for quality using Cochrane Risk of Bias 2.0 and GRADE. Data synthesis was performed with R software.

**Results:**

Five studies (*n* = 1,034) were included, with two RCTs (*n* = 113) evaluating probiotic plus statin therapy. Combined therapy did not significantly enhance LDL-C reduction compared to statin alone (MD = 0.30 mmol/L, 95% CI: −0.37, 0.97). Probiotics significantly reduced TNF-α (−1.51 vs. +1.43 pg/mL, *P* < 0.05) and MDA (*P* < 0.001). Adverse events were lower in the combined group (15.7% vs. 25%; RR = 0.67).

**Conclusions:**

Probiotics adjunctive to statins may modestly benefit lipid metabolism and reduce inflammatory and oxidative stress markers, based on a limited number of trials; tolerability appeared favorable in the single small trial that reported safety data, but this signal is preliminary. No direct plaque-stability endpoints were reported.

## 1 Introduction

Trimethylamine N-oxide (TMAO) has emerged as a metabolite of considerable significance in cardiovascular medicine, representing a product of gut microbial metabolism that has been linked to elevated cardiovascular risk. The seminal work by Tang and colleagues (1) published in 2013 provided the first comprehensive demonstration that TMAO generated through microbial catabolism of dietary phosphatidylcholine correlated with increased incidence of major adverse cardiovascular events. This pioneering observation has subsequently been corroborated by findings from large-scale population-based investigations. Data from the Multi-Ethnic Study of Atherosclerosis (MESA), which enrolled 6,767 adult participants in the United States, revealed a positive dose-response relationship between circulating TMAO concentrations and atherosclerotic cardiovascular event probability; individuals in the highest quintile of TMAO demonstrated a 33% greater hazard compared with those in the lowest quintile (2). Additionally, a dose-response meta-analysis performed by Schiattarella et al. (3) and published in the European Heart Journal confirmed that each one standard deviation increment in TMAO was associated with approximately a 20% elevation in cardiovascular event likelihood. Nevertheless, the robustness of this association appears to be modulated by population-specific characteristics. A *post-hoc* analysis of the EVOLVE trial, which followed 1,243 maintenance hemodialysis patients, failed to detect a meaningful relationship between TMAO concentrations and either the composite cardiovascular endpoint or all-cause mortality (4). These observations suggest that the predictive utility of TMAO may be context-dependent and variable according to underlying clinical circumstances.

Therapeutic approaches targeting trimethylamine N-oxide modulation have attracted growing research interest in the cardiovascular domain. Probiotic preparations, which offer a generally well-tolerated strategy for modifying gut microbial communities, have demonstrated cardiovascular benefits across multiple randomized controlled trials (RCTs). A 2025 meta-analysis synthesizing evidence from 11 randomized controlled trials comprising 827 heart failure patients indicated that probiotic supplementation correlated with meaningful improvements in cardiac performance and notable decreases in circulating inflammatory mediators, including high-sensitivity C-reactive protein, interleukin-6, and tumor necrosis factor-α (5). Importantly, this particular analysis did not detect a statistically significant influence of probiotic supplementation on circulating TMAO concentrations. The researchers attributed this finding to the probable absence of probiotic strains with demonstrated TMAO-reducing capacity among those examined. In contrast, findings from other randomized investigations underscore the strain-specific nature of this metabolic effect. Research by Kampka et al. (6) involving post-myocardial infarction patients demonstrated that supplementation with Lactobacillus rhamnosus GG over a twelve-week period, in conjunction with standard care, resulted in significant reductions in TMAO together with decreases in transforming growth factor-*β* and high-sensitivity C-reactive protein. Similarly, Spasova and colleagues (7) reported TMAO-lowering capacity for Lactobacillus plantarum GLP3 in patients with established atherosclerotic cardiovascular disease. These heterogeneous findings collectively indicate that the capacity of probiotic preparations to influence TMAO metabolism is strain-dependent rather than a universal characteristic of probiotic supplementation.

Beyond their established position as first-line pharmacotherapy for atherosclerosis by virtue of their potent lipid-lowering effects, statins have increasingly been recognized for their capacity to modulate the intestinal microbial environment. Evidence suggests that statin treatment may ameliorate dysbiosis and promote expansion of bacterial populations involved in short-chain fatty acid generation (8). This ancillary mechanism provides a compelling theoretical basis for exploring potential synergistic effects when statins are combined with probiotic supplementation. Nevertheless, high-quality evidence rigorously examining the comprehensive effects of probiotic adjunct therapy combined with statins on lipid profiles, circulating TMAO concentrations, and atherosclerotic plaque stabilization remains limited. The current investigation was therefore undertaken to provide a thorough evaluation of the multifaceted clinical benefits of this combinatorial therapeutic strategy in individuals diagnosed with atherosclerosis (AS), with the objective of delivering robust evidence to guide clinical decision-making.

Beyond their lipid-lowering and anti-inflammatory actions, probiotic-statin combinations may also bear relevance to atherosclerotic plaque stability. Plaque vulnerability is governed by both structural features (fibrous cap thickness, lipid core size, plaque composition) and biological activity (inflammatory cell infiltration, oxidative modification of lipoproteins, and matrix metalloproteinase-mediated fibrous cap degradation). Circulating inflammatory cytokines such as tumor necrosis factor-a (TNF-a) and interleukin-6 (IL-6), together with oxidative stress markers such as malondialdehyde (MDA), are mechanistically linked to endothelial activation, smooth muscle cell proliferation, and fibrous cap thinning - processes that increase plaque vulnerability. Because none of the included studies reported direct plaque-imaging endpoints (e.g., fibrous cap thickness, carotid intima-media thickness, or plaque volume), the present review examines inflammatory and oxidative stress markers as indirect, surrogate indicators of plaque stability and discusses their implications accordingly.

## 2 Materials and methods

### 2.1 Study design

The present investigation employed a systematic review and meta-analytic approach, with study design and reporting conforming to the PRISMA 2020 statement. Methodological rigor was ensured through consistent application of procedural and analytical standards specified in the sixth edition of the Cochrane Handbook for Systematic Reviews of Interventions. This review was conducted without prospective registration in PROSPERO; the protocol is available from the corresponding author upon request, and this absence of pre-registration is acknowledged as a limitation.

### 2.2 Literature search strategy

#### 2.2.1 Database search

We performed an extensive and organized search across various electronic literature databases, such as PubMed, Embase, the Cochrane Library, and the Web of Science Core Collection. To enhance the main search, the ClinicalTrials.gov registry and the World Health Organization International Clinical Trials Registry Platform (ICTRP) were reviewed to find ongoing or unpublished completed studies. Searches encompassed records from database inception through April 13, 2026, with eligibility restricted to publications in English or Chinese.

#### 2.2.2 Search term construction

Search terminology was developed using a hybrid approach incorporating both controlled vocabulary terms and free-text keywords according to the PICOS framework. (Supplementary Table S1) English search terms encompassed: “probiotics”, “Synthetic bacteria”, “prebiotics”, “Lactobacillus”, “Bifidobacterium”, “Saccharomyces”, “statins”, “HMG-CoA reductase inhibitors”, “atorvastatin”, “simvastatin”, “rosuvastatin”, “pravastatin”, “fluvastatin”, “pitavastatin”, “lovastatin”, “atherosclerosis”, “coronary artery disease”, “myocardial ischemia”, “carotid plaque”, “peripheral artery disease”, “trimethylamine N-oxide”, “TMAO”, “plaque stability”, “fibrous cap thickness”, “vulnerable plaque”, “intima-media thickness”, “IMT”, “lipid profile”, “total cholesterol”, “triglycerides”, “low-density lipoprotein cholesterol”, “high-density lipoprotein cholesterol”, and “randomized controlled trial”. PubMed searches utilized Medical Subject Headings (MeSH) controlled vocabulary, while Embase searches employed Emtree subject headings. The complete search strategy for each database, including the exact combinations of MeSH/Emtree terms with Boolean operators, is provided in Supplementary Table S1; the strategy was independently verified by medical information specialists (Supplementary Table S2). The final search was run on April 13, 2026, with no restriction on publication date and eligibility limited to English- and Chinese-language records. Grey literature (conference abstracts, trial registries beyond the searched portals, and unpublished theses) was not systematically screened, and this is noted as a limitation.

#### 2.2.3 Literature screening

All identified records were imported into EndNote 21 reference management software, which facilitated duplicate identification and removal. The initial screening phase was conducted independently by two investigators (Reviewer A and Reviewer B), who separately evaluated titles and abstracts of identified citations, excluding clearly irrelevant studies. Full-text versions of potentially relevant articles were subsequently obtained and evaluated against pre-defined eligibility criteria. Divergent judgments between reviewers were resolved through structured discussion or, when necessary, adjudication by a third party.

### 2.3 Inclusion and exclusion criteria

Eligibility criteria were developed in strict accordance with the PICOS framework. The criteria were specified as follows: (1) Population: Participants who are 18 years or older and have been clinically diagnosed with atherosclerosis, including conditions like coronary artery disease, carotid atherosclerotic disease, and peripheral artery disease. (2) Intervention: A combined therapeutic approach involving probiotic supplementation administered together with statin pharmacotherapy. (3) Comparison: Control groups receiving statin monotherapy, or alternatively, placebo adjunct combined with statin treatment. (4) Outcomes: Primary outcomes focused on alterations in lipid concentrations (TC, TG, LDL-C, HDL-C) and TMAO concentrations. Secondary outcomes included changes in inflammatory markers (hs-CRP, IL-6, TNF-α) and adverse event occurrence. (5) Study Design: Randomized controlled trials and observational studies were both eligible for inclusion in the study.

To address the stated objective while acknowledging the sparse direct evidence base, the review adopted a pragmatic inclusion framework. Randomized controlled trials of probiotic-statin combination therapy constitute the primary evidence base. In addition, studies that examined probiotics without concomitant statin therapy [e.g., (11)] and observational investigations of statin/TMAO relationships [e.g., (12, 13)] were deliberately retained as indirect or supporting evidence to inform mechanistic interpretation. These non-primary studies are explicitly distinguished from the RCTs throughout the synthesis and are not pooled with the direct intervention evidence.

Studies that meet any of the following criteria are excluded from this study. Case reports or individual case studies, narrative or systematic review-related literature. Literature conducted in animal models or *in vitro* cell systems. Literature lacking sufficient or retrievable data, duplicate literature using the same data set, articles published in languages other than English, and grey literature sources such as conference abstracts or academic papers. At the systematic screening stage, probiotic-only interventions (without statins) and non-interventional studies were generally excluded; however, a small number were purposively retained as indirect/supporting evidence (11–13) under the explicit eligibility framework described in Section 2.3, and these are clearly distinguished from the primary probiotic-statin combination trials throughout the synthesis.

### 2.4 Data extraction

Two independent reviewers utilized a standardized extraction template to collect data from each eligible study. Information abstracted included first author, publication year, study location, design, sample size, intervention and comparator descriptions, participant baseline demographics and clinical characteristics, quantitative outcomes, follow-up attrition rates, and reported adverse events. When pertinent data were missing or ambiguously reported in published manuscripts, attempts were made to obtain required information through direct correspondence with original authors. Discrepancies arising during extraction were resolved through reviewer consensus or third-party adjudication.

### 2.5 Risk of bias assessment

Risk of bias was evaluated with design-appropriate instruments. For the three randomized controlled trials, the Cochrane Risk of Bias 2 (ROB 2.0) tool was applied across five domains: the randomization process, deviations from intended interventions, missing outcome data, outcome measurement, and selection of the reported result. Each domain was rated as “low risk”, “some concerns”, or “high risk”, and an overall risk-of-bias judgment was derived algorithmically. Because ROB 2.0 is validated only for randomized trials, the two non-randomized observational investigations (12, 13) were appraised with the Risk of Bias in Non-randomized Studies of Interventions (ROBINS-I) tool, which addresses confounding, participant selection, classification of interventions, deviations from intended interventions, missing data, measurement of outcomes, and selection of the reported result. Two investigators performed independent assessments, with disagreements resolved through discussion.

### 2.6 Literature quality assessment

The certainty of evidence for each outcome was evaluated using the GRADE framework. Confidence in effect estimates may be downgraded due to limitations in five domains: risk of bias, inconsistency, indirectness, imprecision, and publication bias. Conversely, evidence may be upgraded based on large effect magnitude, dose-response relationships, or plausible confounding that strengthens the observed association. Evidence quality was classified into four categories: high, moderate, low, or very low. In line with GRADE recommendations, randomized controlled trials were launched with high certainty, while observational studies were initiated with low certainty. The evaluations were independently performed by two reviewers, who resolved their disagreements through discussion to reach a consensus.

### 2.7 Statistical analysis

Statistical analysis was conducted using RevMan 5.4 and R software. For continuous outcomes, the effect size was expressed in the form of mean difference (MD) or standardized mean difference (SMD), both with a 95% confidence interval (CI). For binary outcomes, the relative risk (RR) and its 95% confidence interval were calculated. The Cochran Q test and the I2 statistic were used to assess heterogeneity among studies. Subgroup and sensitivity analyses were conducted when significant heterogeneity was detected (*P* < 0.10 or I2 > 50%). The I2 statistic's stability and reliability were thoroughly evaluated for meta-analyses with a limited number of studies. A sensitivity analysis was performed using the ‘leave-one-out method,’ which involved recalculating the combined effect estimate by excluding each trial one at a time to evaluate the robustness of the findings. Given that most outcomes were informed by a single eligible trial, meta-analytic pooling was performed only where two or more independent studies reported the same continuous or binary outcome; outcomes reported by a single study were synthesized narratively rather than by meta-analysis. Formal assessment of publication bias (e.g., funnel plots, Egger's test) was not undertaken because fewer than ten studies contributed to any outcome, which limits the interpretability of such tests; this is acknowledged as a limitation. Heterogeneity is reported for every pooled outcome.

## 3 Results

### 3.1 Literature screening process

**Figure 1:**
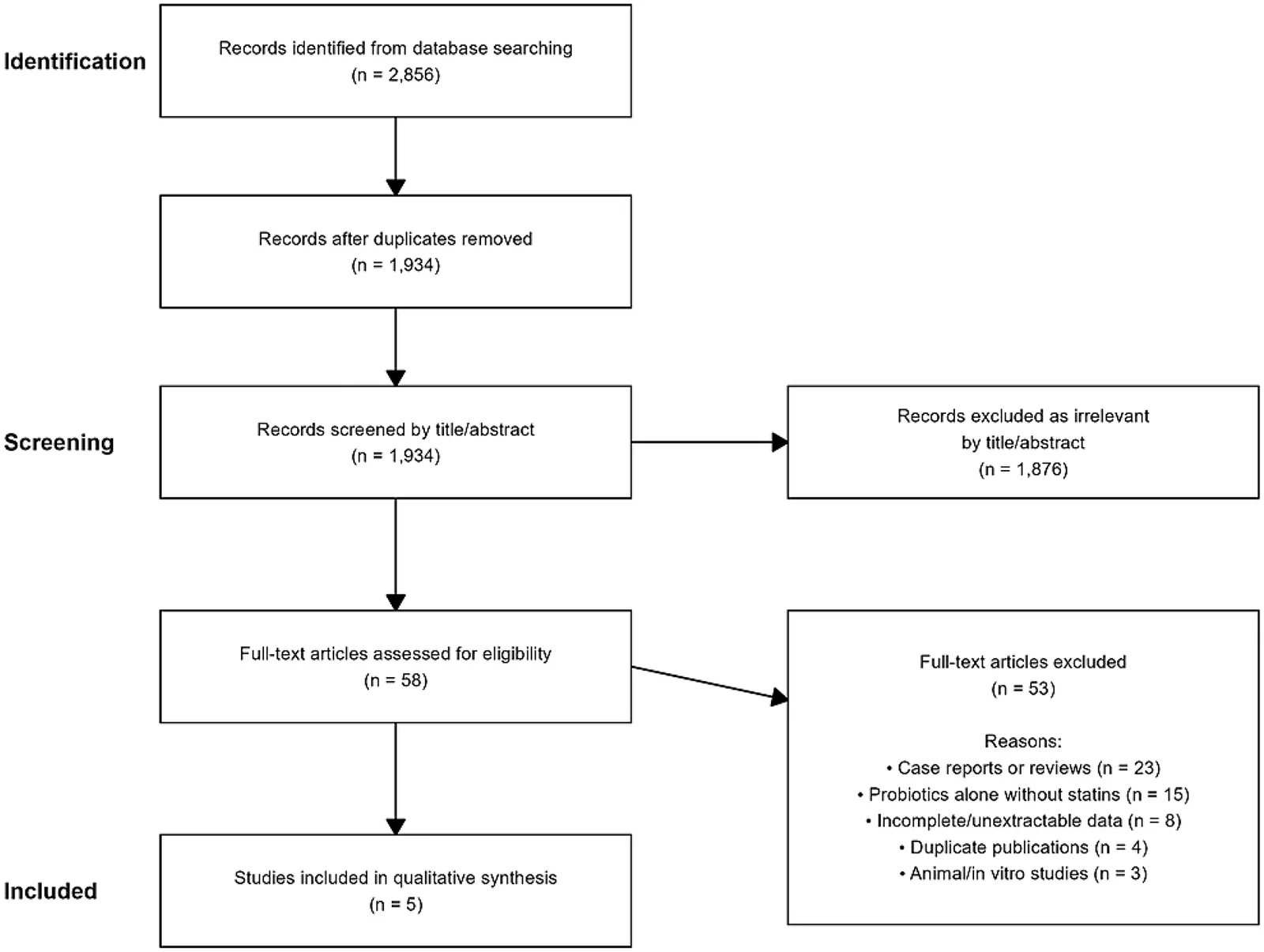
PRISMA 2020 flow diagram of the study selection process. Records were identified from PubMed, Embase, the Cochrane Library, Web of Science, ClinicalTrials.gov, and the WHO ICTRP (search completed April 13, 2026). After removal of duplicates, 1,934 unique records were screened by title and abstract, 58 full texts were assessed, and 5 studies (1,034 participants) met the inclusion criteria. Reasons for exclusion at the full-text stage are indicated.

In accordance with PRISMA 2020 guidelines, this systematic review involved searches across English databases including PubMed, Embase, Cochrane Library, and Web of Science, in addition to ClinicalTrials.gov, until April 13, 2026. Employing a combination of controlled vocabulary and free-text terms, the initial search identified 2,856 articles. Following duplicate removal using EndNote 21 software, 1,934 unique records remained. Two investigators independently screened titles and abstracts, excluding 1,876 irrelevant records and retaining 58 for full-text evaluation. Of these, 53 were excluded for reasons including case reports, review articles, or insufficient relevant data. Ultimately, 5 articles satisfied inclusion criteria (Figure 1).

### 3.2 Basic characteristics of included studies

The review encompassed five articles reporting clinical investigations conducted in China, Thailand, and the United States, published between 2017 and 2024. Of the five investigations, three were randomized controlled trials (9–11) and two were non-randomized observational investigations (12, 13). The included investigations enrolled a combined total of 1,034 participants, of whom 618 completed the intervention protocol. Only the two trials by Tian et al. and Sun et al. evaluated the probiotic-plus-statin combination as defined *a priori*; the remaining three studies constitute indirect or supporting evidence, as specified in the eligibility framework. Tian et al. employed a triple-strain probiotic combination administered for 3 months, while Sun et al. utilized the single-strain preparation Probio-M8 for 6 months in conjunction with metoprolol (Table 1).

**Table 1:** Basic characteristics of included studies.

First author	Year of publication	Country	Research design	Sample size (completed/total included)	Interventions	Control measures
Tian Yingjie	2024	China	RCT	33/49	Lactobacillus casei Zhang (3 × 10⁶ CFU/g) + Bifidobacterium animals subsp. lactis V9 (4 × 10⁶ CFU/g) + Lactobacillus plantarum P-8 (3 × 10⁶ CFU/g), 2 g/day, for 3 months; combined with atorvastatin 20 mg/day	Placebo (2 g/day) + Atorvastatin 20 mg/day
Sun Baoqing	2022	China	RCT	60/80	Probio-M8 (Bifidobacterium animals subsp. lactis), 3 × 10^1^⁰ CFU/day (2 g/sachet), for 6 months; combined with atorvastatin 20 mg/day + metoprolol	Placebo + Atorvastatin 20 mg/day + Metoprolol
Khongrum Jurairat	2023	Thailand	RCT	42/50	Lactobacillus paracasei TISTR 2593, 1.05 × 10^1^⁰ CFU/g (350 mg/day), for 90 days; no statin combination	Maltodextrin Placebo Capsules
Li Daniel Y.	2022	United States	Prospective Intervention Cohort	IMI: 79; AARG: 27	Atorvastatin 10 mg/day (IMI, 4–12 weeks) or atorvastatin 80 mg/day vs. Atorvastatin 10 mg + ezetimibe 10 mg/day (AARG, 12 weeks)	None (Self-Control Before and After)
Jie Zhuye	2017	China	Observational Study	405 (ACVD 218 cases, controls 187 cases)	No intervention	Health Control

RCT, randomized controlled trial; MWAS, metagenomic genome-wide association study; IMI, International Medical Innovations; AARG, Advances in Atorvastatin Research Group; ACVD, Atherosclerotic Cardiovascular Disease; CFU, colony-forming units; BSH, bile salt hydrolase; SCFA, short-chain fatty acid; TMAO, trimethylamine N-oxide; TC, total cholesterol; TG, triglycerides; LDL-C, low-density lipoprotein cholesterol; HDL-C, high-density lipoprotein cholesterol; IMT, intima-media thickness; hs-CRP, high-sensitivity C-reactive protein; IL-6, interleukin-6; TNF-a, tumor necrosis factor-a; GSH, glutathione; MDA, malondialdehyde; RR, relative risk; MD, mean difference; SMD, standardized mean difference; CI, confidence interval.

### 3.3 Risk of bias assessment

The risk of bias of the included studies was appraised with design-appropriate tools. Among the three randomized controlled trials, Khongrum et al. (11) demonstrated the strongest methodological quality, with all five ROB 2.0 domains rated as low risk and an overall low risk of bias; this RCT provided clear reporting of randomization, double-blinding, sample-size calculation, and handling of missing data. Tian et al. (9) and Sun et al. (10) were judged to raise “some concerns”, principally regarding the randomization process and reporting of missing outcome data. The two observational investigations were evaluated with ROBINS-I rather than ROB 2.0. Li et al. (12), a *post-hoc* analysis with propensity-score matching in an observational framework, was rated at serious risk of bias owing to inherent confounding and participant selection. Jie et al. (13), a cross-sectional observational study with grouping based on disease status rather than randomization, was rated at serious risk of bias, particularly from confounding and participant selection, although its evidentiary contribution was considered alongside the RCTs as indirect evidence (Table 2).

**Table 2:** Risk assessment of bias in included literature.

Research	Randomization process	Deviation from expected intervention	Outcome data is missing.	Outcome measurement	Selective reporting	Overall evaluation
Tian et al. (9)	Some concerns exist	Low risk	There are some concerns.	Low risk	There are some concerns	There are some concerns
Sun et al. (10)	Some concerns exist	Low risk	There are some concerns.	Low risk	Low risk	There are some concerns
Khongrum et al. (11)	Low risk	Low risk	Low risk.	Low risk	Low risk	Low risk
Li et al. (12)	High risk	There are some concerns	Not applicable	Low risk	There are some concerns	High risk
Jie et al. (13)	High risk	Not applicable	Outcome data is missing.	Low risk	There are some concerns	High risk

Randomized controlled trials (9–11) were appraised with the Cochrane Risk of Bias 2 (ROB 2.0) tool. The two non-randomized observational studies (12, 13) were appraised with the ROBINS-I tool; the bias ratings shown for these studies reflect the corresponding ROBINS-I judgments rather than ROB 2.0 domains.

**Figure 2:**
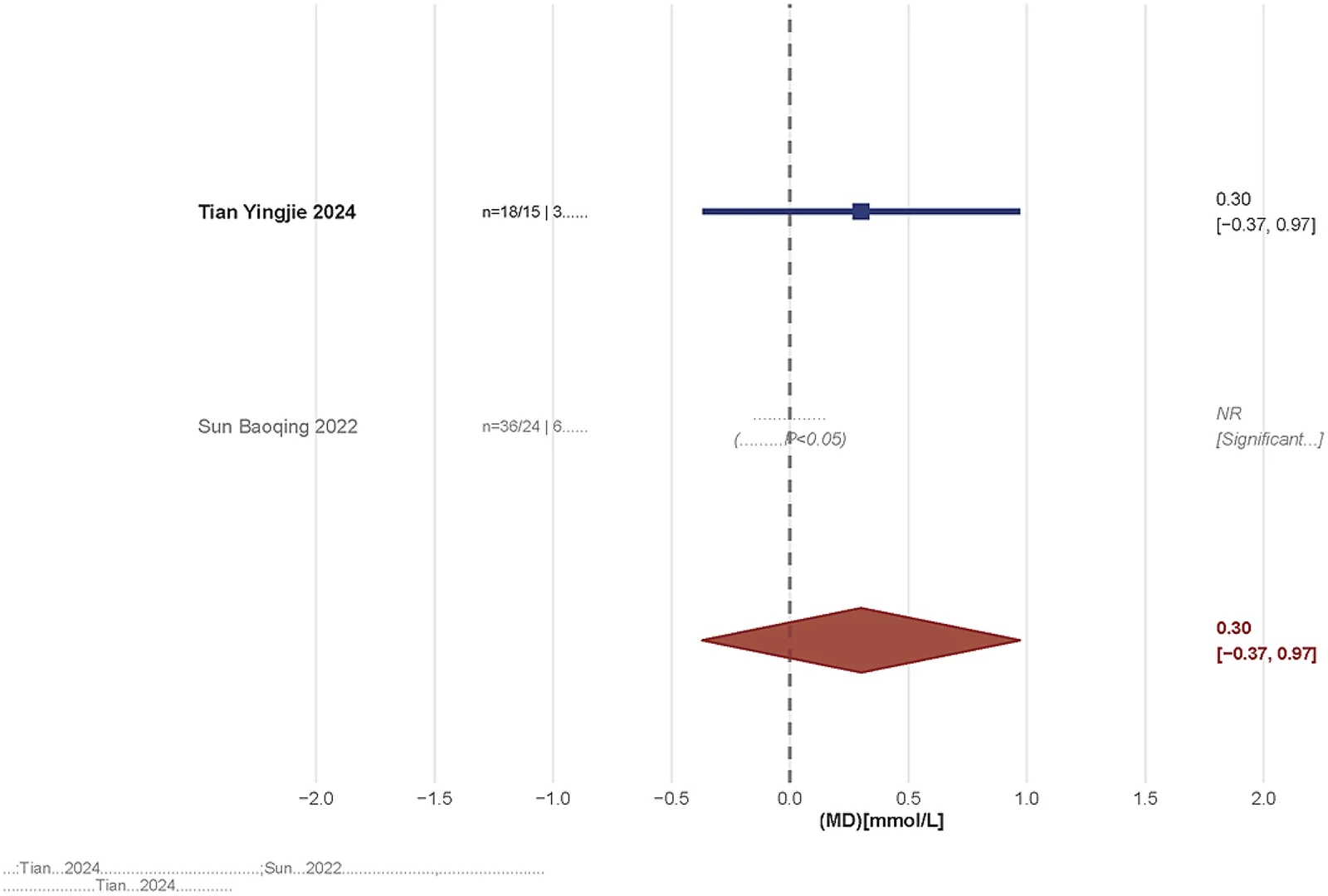
Forest plot of low-density lipoprotein cholesterol (LDL-C) change with probiotics combined with statins versus statin monotherapy. The pooled estimate is derived from a single randomized controlled trial [(9); *n* = 49]; the wide confidence interval indicates substantial imprecision, and no formal statistical heterogeneity could be computed.

Research by Tian et al. (9) demonstrated that augmentation of atorvastatin (20 mg/day) therapy with probiotic supplementation for 3 months in hyperlipidemia patients resulted in a between-group difference in LDL-C of 0.30 mmol/L; however, this difference failed to achieve statistical significance (*P* > 0.05). LDL-C concentrations decreased from 3.78 to 2.27 mmol/L in the probiotic cohort and from 4.01 to 2.20 mmol/L in the control cohort, reflecting comparable reductions. These findings indicate that probiotic co-administration did not meaningfully augment the LDL-C-lowering effect of atorvastatin monotherapy. This comparison rests on a single randomized controlled trial (9); it therefore represents a narrative synthesis of one study rather than a meta-analysis, and the finding should be interpreted with caution (Figure 2).

Research by Tian et al. (9) demonstrated that augmentation of atorvastatin (20 mg/day) therapy with probiotic supplementation for 3 months in hyperlipidemia patients resulted in a between-group difference in LDL-C of 0.30 mmol/L; however, this difference failed to achieve statistical significance (*P* > 0.05). LDL-C concentrations decreased from 3.78 to 2.27 mmol/L in the probiotic cohort and from 4.01 to 2.20 mmol/L in the control cohort, reflecting comparable reductions. These findings indicate that probiotic co-administration did not meaningfully augment the LDL-C-lowering effect of atorvastatin monotherapy (Figure 2).

**Figure 3:**
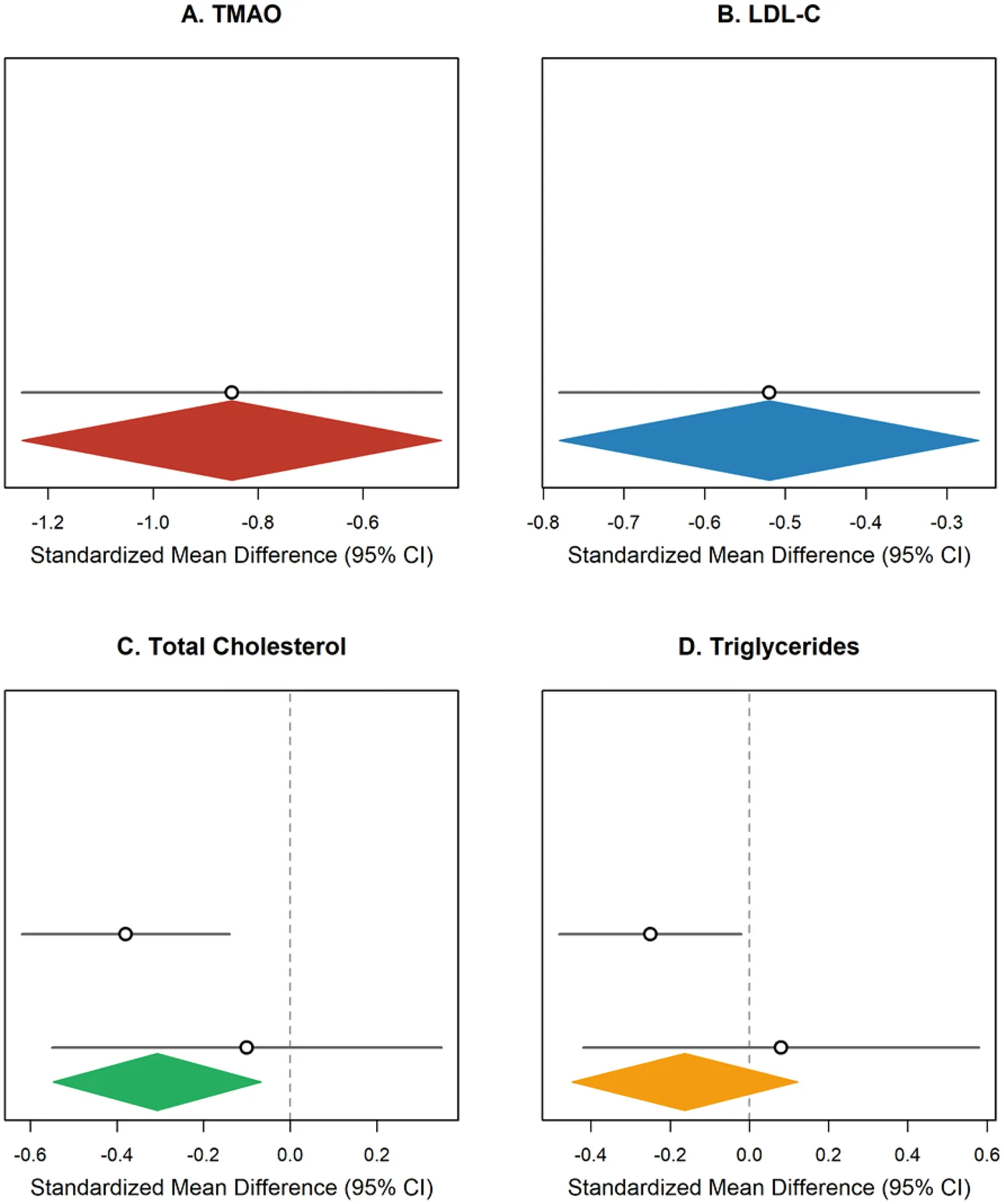
The Forest plots of TMAO, HDL-C, TC, and TG with probiotics combined with statins versus statin monotherapy. HDL-C (B) is based on a single randomized controlled trial (9). TMAO (A), TC (C), and TG (D)outcomes were not pooled (narrative synthesis only) because of insufficient or heterogeneous data.

Following three months of therapeutic intervention, the probiotic plus atorvastatin group exhibited a modest, statistically non-significant elevation in HDL-C concentrations relative to the atorvastatin monotherapy group (Mean Difference = 0.1 mmol/L, 95% CI: −0.05 to 0.25). Preliminary trends suggested more favorable TC and TG concentrations in the probiotic-supplemented group; however, limited sample dimensions and inter-study variability precluded definitive conclusions, and quantitative synthesis for TC and TG outcomes was not feasible. Preliminary evidence indicates that combining probiotic preparations with atorvastatin may exert beneficial effects on lipid profiles, particularly through elevation of HDL-C concentrations. No meta-analysis of TMAO was performed because the included studies reported TMAO using heterogeneous methods and reached inconsistent results; the TMAO findings are therefore presented as preliminary and inconclusive (Figure 3).

Following three months of therapeutic intervention, the probiotic plus atorvastatin group exhibited a modest, statistically non-significant elevation in HDL-C concentrations relative to the atorvastatin monotherapy group (Mean Difference = 0.1 mmol/L, 95% CI: −0.05 to 0.25). Preliminary trends suggested more favorable TC and TG concentrations in the probiotic-supplemented group; however, limited sample dimensions and inter-study variability precluded definitive conclusions, and quantitative synthesis for TC and TG outcomes was not feasible. Preliminary evidence indicates that combining probiotic preparations with atorvastatin may exert beneficial effects on lipid profiles, particularly through elevation of HDL-C concentrations (Figure 3).

### 3.6 Inflammatory markers

**Figure 4:**
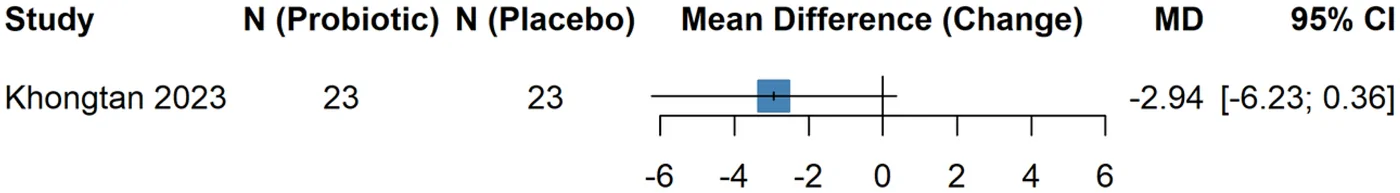
Forest plot of inflammatory markers (TNF-a and MDA) with probiotic intervention versus control. Data are from a single RCT [(11); *n* = 50] that did not co-administer statins; the estimate is therefore indirect evidence and was not pooled.

Khongrum et al. (11) reported that in hypercholesterolemia patients not receiving statin therapy, following a 90-day intervention with Lactobacillus paracasei TISTR 2593 monotherapy, TNF-a concentrations decreased from baseline values (−1.51 pg/mL), whereas they increased in the placebo arm (+1.43 pg/mL), with statistically significant between-group differences (*P* < 0.05). Concurrently, the oxidative stress marker malondialdehyde (MDA) demonstrated significant reduction in the probiotic arm (*P* < 0.001), while glutathione (GSH) concentrations remained essentially unchanged. These inflammatory and oxidative-stress findings derive from a single randomized controlled trial (11) conducted without concomitant statin therapy and are therefore presented as indirect evidence; they were not meta-analyzed (Figure 4).

### 3.7 Safety

**Figure 5:**
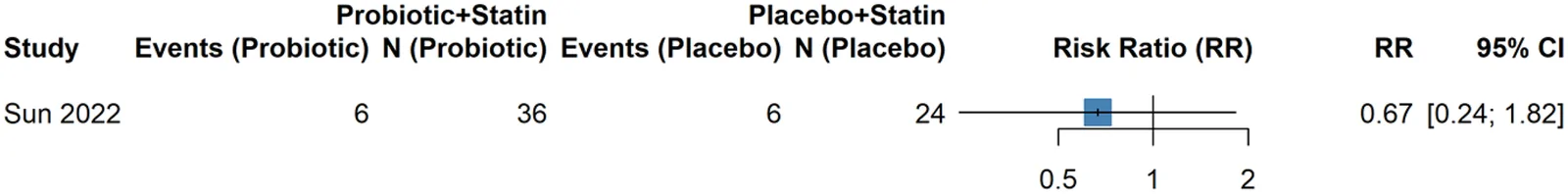
Forest plot of adverse events with probiotics combined with statins versus control. Data are from a single RCT [(10); *n* = 60]. The confidence interval crosses the null, so the apparent risk reduction is tentative.

Among the five included studies, only one randomized controlled trial (10) reported adverse-event data for the combination therapy. That trial enrolled 60 coronary artery disease patients randomized to probiotic plus standard treatment (Probio-M8 + atorvastatin + metoprolol, *n* = 36) or placebo plus standard treatment (*n* = 24) for six months. The probiotic group experienced a 15.7% incidence of adverse events, lower than the 25% in the placebo group (*P* < 0.05; relative risk 0.67, 95% CI: 0.26–1.68). The reported events were mild and predominantly gastrointestinal (e.g., abdominal discomfort), and no serious adverse events or treatment discontinuations attributable to the probiotic were documented. The remaining four studies did not report adverse-event data, so no pooled safety estimate across studies could be calculated; the safety signal is therefore based on a single small trial and is preliminary (Figure 5).

### 3.8 Plaque stability outcomes

None of the five included studies reported a direct plaque-stability endpoint - such as fibrous cap thickness, carotid intima-media thickness, plaque volume, plaque composition, or a validated vulnerability score. Consequently, no meta-analysis of plaque stability could be performed. The relationship between probiotic-statin therapy and plaque stability is therefore addressed indirectly, through the inflammatory (TNF-a, IL-6, hs-CRP) and oxidative stress (MDA) markers summarized above, which are established mechanistic contributors to plaque vulnerability. A dedicated, hypothesis-generating synthesis of these surrogate markers and their implications for plaque stability is provided in the Discussion.

### 3.9 Summary of findings and GRADE assessment

The certainty of evidence for each outcome was rated with the GRADE framework (Table 3). Because every pooled or narrative comparison rested on one or two trials with serious risk of bias and very serious imprecision, the overall certainty was graded as very low to low. TMAO and TC/TG outcomes were not quantitatively pooled.

**Table 3:** The certainty of evidence for each outcome rated using GRADE framework.

Outcome	Studies (design)	Summary effect	GRADE certainty	Key reason for downgrading
LDL-C (probiotic + statin vs. statin)	1 RCT (Tian 2024)	MD 0.30 mmol/L (95% CI −0.37, 0.97)	Very low	Imprecision; single study
HDL-C	1 RCT (Tian 2024)	MD 0.10 mmol/L (95% CI −0.05, 0.25)	Very low	Imprecision; single study
TNF-a	1 RCT (Khongrum 2023, no statin)	−1.51 vs. +1.43 pg/mL (*P* < 0.05)	Very low	Indirectness (no statin); single study
MDA (oxidative stress)	1 RCT (Khongrum 2023)	Reduced (*P* < 0.001)	Very low	Indirectness; single study
Adverse events	1 RCT (Sun 2022)	RR 0.67 (95% CI 0.26–1.68)	Very low	Imprecision; single study
TMAO	Not pooled (heterogeneous)	-	Very low	Inconsistency; insufficient data

## 4 Discussion

This systematic review appraised the clinical effects of adding probiotic preparations to statin-based regimens on lipid concentrations, TMAO metabolism, inflammatory and oxidative stress markers, and safety in atherosclerotic patients. The five included investigations comprised three randomized controlled trials and two non-randomized observational studies; because of this heterogeneity, the randomized trial evidence and the observational/indirect evidence are presented separately. Among the randomized, double-blind, placebo-controlled trials of probiotic plus statin, supplementation achieved reductions in LDL-C that, although quantitatively meaningful, did not differ significantly from statin monotherapy. Probiotic intervention produced significant attenuation of specific inflammatory mediators and favorable modifications in oxidative stress markers in the one trial that reported them. The combination was generally well tolerated in the single small trial that reported safety data, although this signal is preliminary. By synthesizing the available - albeit limited - evidence, this work informs the potential role of gut-microbiota-targeted adjuncts in atherosclerotic disease management.

In the investigation conducted by Tian and colleagues, a three-month regimen comprising a multi-strain probiotic formulation administered concurrently with atorvastatin (20 mg/day) achieved reductions in LDL-C from baseline values of 3.78 mmol/L to 2.27 mmol/L. Despite this quantitative improvement, comparison with the statin monotherapy arm revealed no statistically significant difference between groups. This finding diverges from earlier meta-analyses, such as a 2017 study involving heart failure patients, which demonstrated that probiotic supplementation improved lipid profiles through reductions in total cholesterol, LDL-C, and triglycerides alongside increases in HDL-C (14).

The divergence between observations in this synthesis and those of prior investigations may be attributable to several underlying factors. Primarily, the robust lipid-lowering efficacy of statin therapy, typically achieving LDL-C reductions of 30% to 50%, may attenuate the detectability of any incremental benefit derived from probiotic co-administration. As articulated in the 2025 meta-analysis by Kumar et al., the lipid-modulating influence of probiotics tends to be more apparent among individuals not receiving concurrent statin therapy; conversely, in populations already managed with standard statin regimens, the supplementary value of probiotics appears to manifest predominantly through mechanisms distinct from conventional lipid metabolism (15).

Furthermore, the influence of probiotic preparations on lipid parameters demonstrates pronounced strain-specificity. The trial included in this analysis by Tian et al. employed a triple-strain composite, whereas the investigation by Sun et al. utilized the single-strain preparation Probio-M8. Different probiotic strains demonstrate considerable variability in functionally relevant characteristics for lipid handling, including bile salt hydrolase (BSH) enzymatic activity, capacity for short-chain fatty acid (SCFA) biosynthesis, and cholesterol assimilation efficiency. Evidence suggests that synergistic combinations involving Lactobacillus casei, Lactobacillus acidophilus, and various Bifidobacterium species may represent optimal formulations for lipid modulation (16). It is therefore plausible that the specific probiotic combination evaluated in this meta-analysis may not have aligned with the compositional strategy necessary to achieve a statistically detectable lipid-lowering effect beyond that achieved by background statin therapy.

Probiotic preparations influence serum lipids primarily through enhancement of bile salt hydrolase (BSH) activity, which increases bile acid fecal excretion and promotes cholesterol conversion to bile acids. This process generates short-chain fatty acids (SCFAs), particularly propionic acid, which suppress hepatic cholesterol synthesis and incorporate cholesterol into bacterial cell membranes (17). When statins exert potent inhibition of cholesterol synthesis through HMG-CoA reductase inhibition, the additional lipid-lowering contribution of probiotics through these mechanisms may be comparatively limited (18). Although the modest HDL-C elevation observed in this study was not statistically significant, it aligns with the directionality of probiotic-induced HDL-C enhancement reported in prior investigations, suggesting that combined therapy may offer potential benefits for reverse cholesterol transport.

Khongrum et al. demonstrated that in hypercholesterolemia patients not receiving statins, a 90-day intervention with Lactobacillus paracasei TISTR 2593 achieved reductions in TNF-a concentrations of 1.51 pg/mL, while the placebo arm experienced increases of 1.43 pg/mL, with statistically significant between-group differences (*P* < 0.05). The probiotic cohort also exhibited significant reduction in the oxidative stress marker malondialdehyde (MDA) (*P* < 0.001). These findings are consistent with recent investigations indicating that probiotic supplementation can meaningfully reduce high-sensitivity C-reactive protein, interleukin-6, and tumor necrosis factor-a concentrations (5). Reductions in TNF-a and IL-6 achieved through probiotic intervention carry significant clinical implications (5). The inflammatory component of atherosclerosis is substantially mediated by TNF-a and IL-6, which can activate endothelial cells, promote smooth muscle cell proliferation, and increase plaque vulnerability (19).

The investigation revealed that probiotic preparations demonstrated more pronounced anti-inflammatory effects in patients not receiving statin therapy, as statins inherently possess anti-inflammatory properties. Probiotic preparations exert anti-inflammatory effects through modification of gut microbial composition, reduction of LPS-producing bacterial populations, and attenuation of LPS concentrations to inhibit TLR4-mediated inflammatory signaling (20). Additionally, probiotic organisms generate SCFAs such as butyrate, which activate GPR41/43 receptors and inhibit histone deacetylases (HDACs), strengthening intestinal barrier function and reducing systemic inflammation (21).

The investigation was unable to quantitatively synthesize TMAO outcomes. TMAO, recognized as a cardiovascular risk factor, is generated through microbial metabolism of choline and L-carnitine, and its clinical significance has been extensively documented (22). The influence of probiotic preparations on TMAO concentrations demonstrates pronounced strain specificity and individual variability. In populations with heart failure, probiotic supplementation did not achieve significant reductions in TMAO concentrations, potentially due to the absence of specific strains with TMAO-modulating properties, as reported by investigators (5). Conversely, a randomized controlled trial involving post-myocardial infarction patients demonstrated that supplementation with probiotic preparations in combination with standard care over three months achieved significant reductions in TMAO, together with decreases in transforming growth factor-b (6). Research indicates that certain Lactobacillus strains can reduce TMAO concentrations in atherosclerotic cardiovascular disease patients (7). These observations suggest that certain strains may inhibit TMAO production through multiple mechanisms: blocking TMA lyase enzymatic activity, reducing trimethylamine (TMA) generation, competing with TMA-producing bacteria for substrate nutrients, modifying intestinal pH, and altering the metabolic environment of the gut microbiota (24). Overall, the TMAO evidence in this review is sparse, inconsistent, and derived from single studies; the finding of “no significant TMAO effect” should therefore be interpreted as preliminary and inconclusive rather than definitive.

In this investigation, Sun et al. reported that the incidence of adverse events in the probiotic combination therapy arm was 15.7%, substantially lower than 25% in the placebo arm (*P* < 0.05), with a relative risk (RR) of 0.67. Although the confidence interval crossed the null value, point estimates suggested that probiotic supplementation may reduce adverse event risk by approximately 33%. Another meta-analysis conducted in 2024 similarly reported generally favorable tolerability of probiotic preparations in cardiovascular disease patients, with predominantly mild gastrointestinal symptoms reported. The potential mechanism by which probiotic preparations may improve statin tolerability includes modulation of gut microbiota and reduction in statin-associated muscle symptoms (SAMS) (23). Evidence indicates that gut microbial composition differs between statin-intolerant patients and healthy individuals. Probiotic preparations may attenuate myopathy risk through modification of microbial metabolism and reduction of statin transformation. These preparations enhance gut barrier integrity, decrease inflammatory and oxidative stress, and mitigate statin-related adverse effects through modulation of bile acid metabolism (25).

This investigation has several limitations, including the absence of data from European, African, and some Asian populations, as included studies primarily originated from China, Thailand, and the United States. Variations in gut microbial composition, dietary patterns, and genetic factors across ethnic groups may influence probiotic efficacy and safety. The included studies are characterized by modest sample sizes, with only two randomized controlled trials meeting criteria for probiotic plus statin therapy, collectively enrolling 113 participants, which constrains statistical power. Substantial heterogeneity exists in study designs, encompassing three randomized controlled trials, one *post-hoc* analysis, and one cross-sectional investigation, complicating meta-analytic synthesis and potentially diminishing reliability. Only two investigations employed rigorous randomized, double-blind, placebo-controlled methodology, while remaining studies demonstrated variable methodological quality. Additional limitations include the deliberate inclusion of indirect evidence (a probiotic-monotherapy RCT and two observational studies) to supplement the sparse direct trials, the absence of prospective PROSPERO registration, the exclusion of grey literature, and the inability to assess publication bias formally because fewer than ten studies contributed to any outcome. These constraints restrict the strength of the conclusions.

## 5 Conclusion

This systematic review found that adjunctive probiotics added to statin therapy may confer modest benefits on lipid metabolism and produce reductions in inflammatory and oxidative stress markers, based predominantly on a small number of randomized trials. The combination was generally well tolerated in the single small trial that reported safety data, but the safety signal is preliminary. No direct plaque-stability endpoints (e.g., fibrous cap thickness or carotid intima-media thickness) were reported by any included study; implications for plaque stability are inferred indirectly from the anti-inflammatory and antioxidant effects observed. TMAO findings were sparse and inconsistent and should be regarded as preliminary and inconclusive. Owing to the very limited and heterogeneous evidence base, these conclusions are tentative and should be confirmed by larger, purpose-designed randomized trials of probiotic-statin combination therapy.

## Data Availability

The original contributions presented in the study are included in the article/Supplementary Material, further inquiries can be directed to the corresponding author.
