# Supplementary material for: Effects of probiotics combined with statins on blood lipids, TMAO, and inflammatory and oxidative stress markers in patients with atherosclerosis: a systematic review and meta-analysis
Source: Front Cardiovasc Med. 2026 Aug 13;13:1897708. doi: 10.3389/fcvm.2026.1897708 (PMC13519406; doi:10.3389/fcvm.2026.1897708)
Supplement: Supplementary file 1 [file Table1.docx]

Table S1. Core Search Term System Based on PICOS

| Category | Keywords/Free Terms | Logical Combination |
| --- | --- | --- |
| **P** | Atherosclerosis, Arteriosclerosis, Atheroma, Coronary Artery Disease, CAD, CHD, Cardiovascular Disease, Plaque, Carotid Intima-Media Thickness, cIMT | OR |
| **I** | Probiotics, Probiotic, Synbiotics, Synbiotic, Lactobacillus, Bifidobacterium, Bifidobacteria, Saccharomyces, Streptococcus thermophilus, Lactic Acid Bacteria, Fermented Milk, Yogurt, Kefir, Gut Microbiota Modulation, Microbiome Therapy | OR |
| **I₂** | Statins, Statin, HMG-CoA Reductase Inhibitors, Atorvastatin, Rosuvastatin, Simvastatin, Pravastatin, Fluvastatin, Pitavastatin, Lovastatin, Hydroxymethylglutaryl-CoA Reductase Inhibitors | OR |
| **C** | Placebo, Control, Standard Care, Conventional Therapy, Statin Monotherapy, Statin Alone | OR |
| **O** | Lipids, Cholesterol, LDL-C, HDL-C, Triglycerides, TG, Total Cholesterol, TC, VLDL-C, Non-HDL-C, Apolipoprotein B, Apolipoprotein A1, Trimethylamine N-oxide, TMAO, Trimethylamine, TMA, Choline, Carnitine, Betaine, Plaque Stability, Plaque Burden, Plaque Composition, Fibrous Cap, Lipid Core, Plaque Vulnerability, Intima-Media Thickness, IMT, cIMT, Endothelial Function, Flow-Mediated Dilation, FMD, Arterial Stiffness, Pulse Wave Velocity, PWV, Inflammation, CRP, hs-CRP, IL-6, TNF-α | OR |
| **S** | Randomized Controlled Trial, RCT, Controlled Clinical Trial, Randomized, Randomised, Placebo, Random Allocation, Double-Blind, Single-Blind, Clinical Trial | OR |
